# Recurrence Plot-Based Approach for Cardiac Arrhythmia Classification Using Inception-ResNet-v2

**DOI:** 10.3389/fphys.2021.648950

**Published:** 2021-05-17

**Authors:** Hua Zhang, Chengyu Liu, Zhimin Zhang, Yujie Xing, Xinwen Liu, Ruiqing Dong, Yu He, Ling Xia, Feng Liu

**Affiliations:** ^1^ School of Information Technology and Electrical Engineering, University of Queensland, Brisbane, QLD, Australia; ^2^School of Instrument Science and Engineering, Southeast University, Nanjing, China; ^3^Science and Technology on Information Systems Engineering Laboratory, The 28th Research Institute of CETC, Nanjing, China; ^4^First Department of Cardiology, People’s Hospital of Shaanxi Province, Xi’an, China; ^5^Dushuhu Public Hospital Affiliated to Soochow University, Suzhou, China; ^6^Department of Biomedical Engineering, Zhejiang University, Hangzhou, China

**Keywords:** cardiac arrhythmia classification, ECG, recurrence plot, Inception-ResNet-v2, deep learning

## Abstract

The present study addresses the cardiac arrhythmia (CA) classification problem using the deep learning (DL)-based method for electrocardiography (ECG) data analysis. Recently, various DL techniques have been utilized to classify arrhythmias, with one typical approach to developing a one-dimensional (1D) convolutional neural network (CNN) model to handle the ECG signals in the time domain. Although the CA classification in the time domain is very prevalent, current methods’ performances are still not robust or satisfactory. This study aims to develop a solution for CA classification in two dimensions by introducing the recurrence plot (RP) combined with an Inception-ResNet-v2 network. The proposed method for nine types of CA classification was tested on the 1st China Physiological Signal Challenge 2018 dataset. During implementation, the optimal leads (lead II and lead aVR) were selected, and then 1D ECG segments were transformed into 2D texture images by the RP approach. These RP-based images as input signals were passed into the Inception-ResNet-v2 for CA classification. In the CPSC, Georgia, and the PTB_XL ECG databases of the PhysioNet/Computing in Cardiology Challenge 2020, the RP-based method achieved an average F1-score of 0.8521, 0.8529, and 0.8862, respectively. The results suggested the excellent generalization ability of the proposed method. To further assess the performance of the proposed method, we compared the 2D RP-image-based solution with the published 1D ECG-based works on the same dataset. Also, it was compared with two traditional ECG transform into 2D image methods, including the time waveform of the ECG recordings and time-frequency images based on continuous wavelet transform (CWT). The proposed method achieved the highest average F1-score of 0.844, with only two leads of the 12-lead ECG original data, which outperformed other works. Therefore, the promising results indicate that the 2D RP-based method has a high clinical potential for CA classification using fewer lead ECG signals.

## Introduction

Cardiac arrhythmia (CA) is a common cardiovascular disease, and it includes various arrhythmias, such as atrial fibrillation (AF), atrioventricular block, premature atrial contraction (PAC), premature ventricular contraction (PVC), and so on. As a life-threatening risk, the CA affects more than 4.3 million people only in America at a total direct annual healthcare cost of up to $US 67.4 billion, which is a heavy economic burden to society (Tang et al., 2014). Many arrhythmias manifest as sequences of the wave with unusual timing or morphology in electrocardiography (ECG) (De Chazal et al., 2004), and analysis of the inherent features of ECG is the most common technique for diagnosis and classification of CA.

Recently, various machine learning methods have been developed for ECG arrhythmia classification. Using the MIT-BIH arrhythmia database, a mixture-of-experts classifier structure was formed to improve the performance of ECG beat classification, which was based on three popular artificial neural networks. The self-organizing maps and the learning vector quantization algorithms were used to train the classifier, and the mixture-of-experts method was used to classify the ECG beat (Hu et al., 1997).

Based on RR-interval features, heartbeat interval features and ECG morphology features, a linear discriminants framework was proposed to allow the classification and diagnosis of CA into five groups: normal, ventricular ectopic beat (VEB), supraventricular ectopic beat (SVEB), the fusion of normal and VEBS, and unknown types (De Chazal et al., 2004). Using RR interval features and a hierarchical heartbeat classification system, another work detected the VEB based on random projection and support vector machine (SVM) ensemble and then analyzed the SVEB with a positive predictive value of 42.2% (Huang et al., 2014). For the CA prediction by traditional machine learning approaches, a set of handcrafted features was extracted from the ECG dataset and then followed by classifiers, including SVM (Ye et al., 2012), artificial neural network (Ince et al., 2009), linear discriminant framework (de Chazal and Reilly, 2006), etc. However, it requires more specific expertise in ECG, thus generally challenging to further improve the performance in complex CA classification.

Due to the improvement of computing power and the availability of a large number of datasets, deep neural networks have recently been used to perform automatic feature extraction and end-to-end classification of CA. One-dimensional (1D) deep convolutional neural networks (CNNs) have become the mainstream means to address these tasks. A 34-layer 1D CNN was applied to 1D ECG rhythm classification with a dataset recorded with a single-lead wearable monitor, which achieved an optimal performance exceeding cardiologists’ performance (Rajpurkar et al., 2017). A 1D CNN was developed in another work to classify 12 rhythm classes with 91,232 single-lead ECG records. The results demonstrated that the end-to-end deep learning (DL)-based method could achieve the same performance as a cardiologist (Hannun et al., 2019). A method was proposed to classify heart diseases using a 1D CNN based on a modified ECG signal in MIT-BIH, St. Petersburg, and PTB dataset (Hasan and Bhattacharjee, 2019). A 31-layer 1D residual CNN (ResNet) was applied to achieve an optimal accuracy in the classification of five different CA based on two-lead ECG signals (Li et al., 2020). Moreover, recurrent neural networks (RNNs) have a memory that captures information about data history and can model data of arbitrary lengths that were widely used for modeling sequential data. Xiong et al. (2018) proposed a neural network named RhythmNet, which combines the strengths of both 1D CNNs and RNNs to classify four different CAs based on the 2017 PhysioNet/Computing in the Cardiology Challenge dataset. They evaluated the algorithm on 3,658 testing data and obtained an F1 accuracy of 0.82. Long-term and short-term memory (LSTM) is one of the RNN and is widely used in time-series signal analysis, such as classification of ECG signals and speech recognition, and so on. An approach that combined 1D CNN and LSTM was developed to automatically classify six types of ECG signals from the MIT-BIH arrhythmia database (Chen et al., 2019). Besides, three works based on the 1st China Physiological Signal Challenge dataset, using 1D CNN, were combined with LSTM to classify the CA. He et al. (2019) developed a model constitutive of 1D CNN and LSTM to learn local features and global features from raw 12-lead ECG signals to realize the classification and achieved the overall F1-score 0.799. Yao et al. (2019) proposed an attention-based time-incremental CNN, using 1D CNN, LSTM, and attention module to extract both spatial and temporal fusion of information from the raw 12-lead ECG data, which reached an overall classification F1-score of 0.812. Chen et al. (2020) applied a neural network that combined 1D CNN, bidirectional RNN, and attention modules to achieve a median overall F1-score of 0.797 for nine types of CA classification based on 12-lead ECG.

In parallel to the development of DL methods for CA classification based on 1D ECG signals, alternative methods have been proposed to transform the time-series signals into 2D matrices that can be handled by those CNN networks dedicated to processing 2D or multi-dimensional signals in the fields of image processing and computer vision. An automatic algorithm was proposed to detect AF, for which the 1D ECG signals have been converted to 2D time-frequency representations and then processed by the network of 2D CNN and Densenet. The method led to an F1 of 0.82 (Parvaneh et al., 2018). In another work, a signal quality index algorithm along with dense CNNs was developed to distinguish AF based on the dataset of 2017 PhysioNet/CinC Challenge by time-frequency representations of one-lead ECG recordings which achieved an overall F1-score of 0.82 (Rubin et al., 2018). Zhao et al. (2019) proposed a method that combined the modified frequency slice wavelet transform (MFSWT) and CNN to PVC recognition. Using this method, the PVC and non-PVC ECG recordings were modeled to a set of time-frequency images, which were then fed into the CNN as the input signals to process the prediction. It achieved a high accuracy of 97.89% for the PVC recognition. Besides, using 2D grayscale images of each ECG recording as input signals, some studies developed 2D CNN to CA classification based on the MIT-BIH database (Jun et al., 2018; Izci et al., 2019).

As described above, various temporal, morphology, and time-frequency features of ECG data have been considered to study the CA classification. However, for specific complex CA classification, these methods may still offer non-robust and unsatisfactory results, and new techniques are thus required for providing better solutions for clinical use. In this work, a 2D DL-based CA classification method using the recurrence plot (RP) technique (Eckmann et al., 1987) was developed. The RP graphically shows hidden patterns and structural changes in time signals or similarities in patterns across the time series. It has been applied to various applications, including Parkinson’s disease identification (Afonso et al., 2018), heart rate variability evaluation (Marwan et al., 2002; Schlenker et al., 2015), paroxysmal AF prediction (Mohebbi and Ghassemian, 2011), and AF and VF and PAC and PVC prediction (Mathunjwa et al., 2021). Different from other time-series representations, RP may provide a visual mechanism for pattern identification, being suitable for combining with state-of-the-art DL approaches. In this work, whether the RP-based DL framework is appropriate for CA classification was studied.

The contributions of this work include the following: (i) this is a prospective study of using RP for modeling ECG signals with 2D texture images that are processed for DL-based CA classification; (ii) the optimal leads (lead II and lead aVR) were selected as an input signal to classify nine classes of CA, implemented with the 1st China Physiological Signal Challenge 2018 open database, and achieved performance with the average F1-score 0.844; (iii) the Inception-ResNet-v2 network was introduced to extract the characteristics of patterns and structural changes from the 2D RP-based images.

The rest of the paper is organized as follows: the approach and the network architecture are described in section “Methodology,” the experiments are detailed in section “Experiment”, and the conclusions are drawn in section “Conclusion.”

## Methodology

In this work, the classification of the CA problem is modeled as a 2D image classification task using RP-based texture images and the Inception-ResNet-v2 architecture.

### Recurrence Plot

The time series such as ECG signals have typical recurrent behaviors, including periodicities and irregular cyclicities (Debayle et al., 2017), which may be difficult to visualize in the time domain. An RP was proposed to explore the m-dimensional phase space trajectory and to visualize the recurrent behaviors of the time series (Eckmann et al., 1987, 1995).

An RP can be formulated as:

(1) $$R_{i,j}=\theta \left(\varepsilon -||x_{i}-x_{j}||\right),i,j=1,\ldots .,N$$

where *N* is the number of considered states (dots at the time series) *x*_*i*_, ε is a threshold distance, |⋅| is a norm (e.g., Euclidean norm), andθ (.) is the Heaviside function.

θ (.) is defined as:

(2) θ(Z)={0,ifz<0 1,otherwise

The original formulation Equation (1) is considered binary caused by ε the threshold distance. In this paper, an un-thresholded approach proposed by Faria et al. (2016) was adopted to avoid information loss by binarization of the R-matrix, with the Euclidean norm, to obtain an RGB image and to make use of the color information in RP images.

The *R*-matrix can be defined as:

(3) $$R_{i,j}=||x_{i}-x_{j}||,i,j=1,\ldots .,N$$

In the present study, the 1D ECG signals have been converted to 2D RP images as the input signals and then fed into the 2D network for classification.

### Network Architecture

**FIGURE 1:**
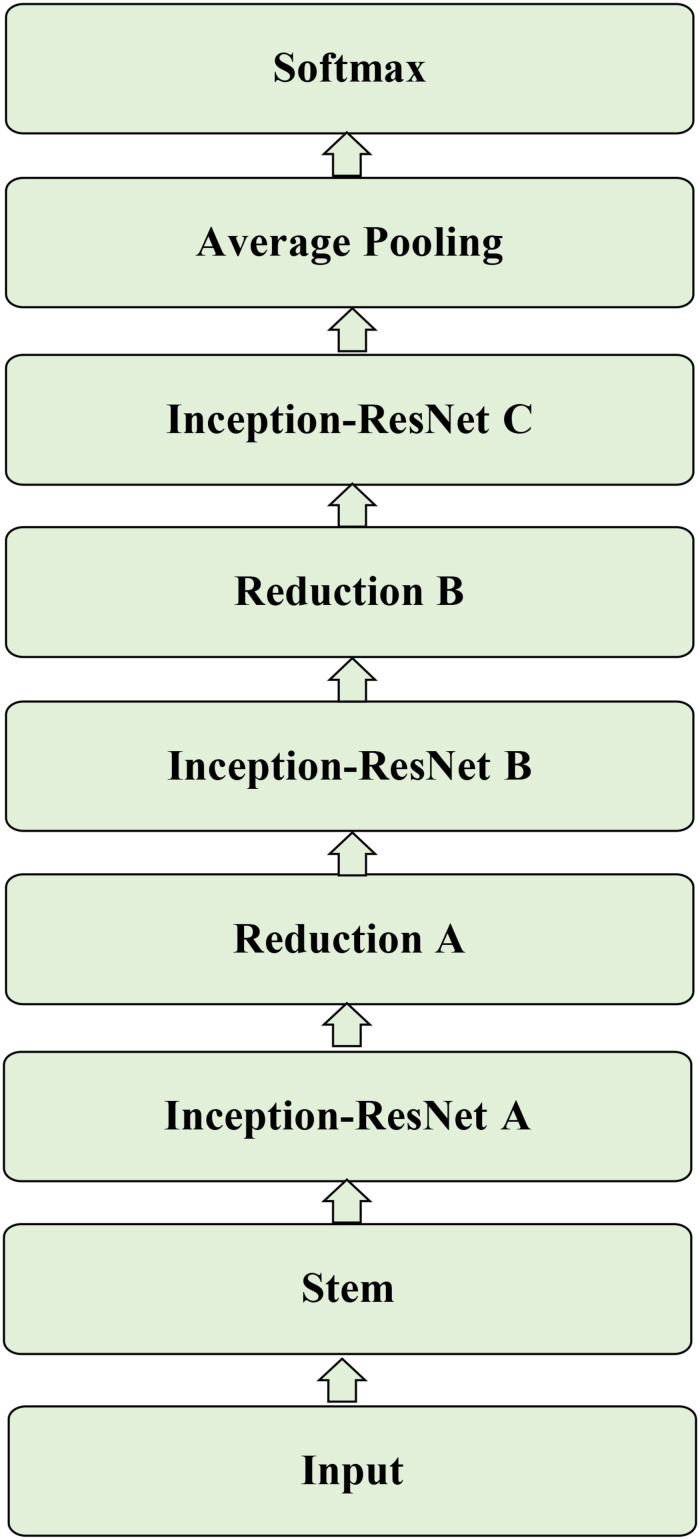
The architecture of the Inception-ResNet-v2.

**FIGURE 2:**
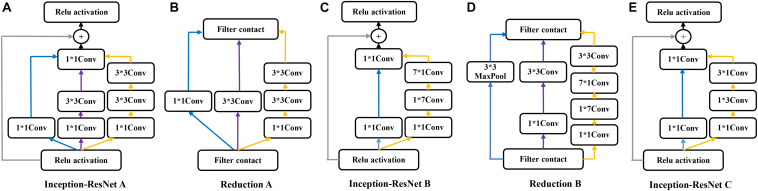
The architecture of the Inception-ResNet A,B,C and Reduction A,B.

The Inception-ResNet-v2 (Szegedy et al., 2016) was used for the CA classification task. The architecture of the network is shown in Figure 1. It contains three parts: the stem is the deep convolutional layers and used to pre-process the original data before entering the Inception-ResNet blocks, including nine layers of convolutional and two max-pooling layers. The second part was detailed in Figure 2. Figure 2A showed the Inception-ResNet-A with two 3 × 3 kernels in the inception module. The Inception-ResNet-B is detailed in Figure 2C with the asymmetric filter combination of one 1 × 7 filter and one 7 × 1 filter in the inception module. The Inception-ResNet-C can be seen in Figure 2E with a small and asymmetric filter combination of one 1 × 3 filter and one 3 × 1 filter; 1 × 1 convolutions were used before the larger filters in these blocks. The network enhances the diversity of the filter patterns by asymmetric convolution splitting. The reduction of A and B in Figures 2B,D was made to increase the dimension, which needs to compensate for the dimensionality reduction caused by the Inception block. The last part is the prediction layer, including pooling and softmax layers.

## Experiment

### ECG Database

The 1st China Physiological Signal Challenge 2018 dataset was used in this study (Liu et al., 2018). The dataset contains 6,877 12-lead ECG recordings lasting from 6 to 60 s for free download, which was labeled the most CA types according to normal sinus rhythm and eight types of arrhythmia: AF, First-degree atrioventricular block (I-AVB), Left bundle branch block (LBBB), Right bundle branch block (RBBB), PAC, PVC, ST-segment depression (STD), and ST-segment elevation (STE). These recordings were collected from 11 Chinese hospitals and sampled at 500 Hz. The dataset details are summarized in Table 1.

**TABLE 1:** Data profile for the ECG dataset.

Type	Recording	Time length (s)				
		Mean	SD	Min	Median	Max
Normal	918	15.43	7.61	10.00	13.00	60.00
AF	1,098	15.01	8.39	9.00	11.00	60.00
I-AVB	704	14.32.	7.21	10.00	11.27	60.00
LBBB	207	14.92	8.09	9.00	12.00	60.00
RBBB	1,695	14.42	7.60	10.00	11.19	60.00
PAC	574	19.46	12.36	9.00	14.00	60.00
PVC	653	20.21	12.85	6.00	15.00	60.00
STD	826	15.13	6.82	8.00	12.78	60.00
STE	202	17.15	10.72	10.00	11.89	60.00
Total	6,877	15.79	9.04	6.00	12.00	60.00

#### Data Splitting and Augmentation

The investigated ECG dataset has two problems. Firstly, the range of each recording length varies from 6 to 60 s. It is not convenient for training the model with non-identical lengths of the ECG recordings. We down-sampled the raw ECG signals to 200 Hz, and then the ECG recordings were segmented into a span of 5 s. Secondly, as demonstrated in Table 1, the dataset is unbalanced, which brings challenges to the classification of arrhythmias. To make it balanced, based on the number of RBBB, the other eight types of data were augmented. For instance, if the duration of the Normal, AF, and STD ECG recordings is over 10 s, the recording were segmented into two 5 s long patches. In this way, 1,836 5 s segments of Normal, 2,195 segments of AF, and 1,651 segments of STD were obtained. For I-AVB, PAC, and PVC recordings, if the duration of the data is over 15 s, it was then divided into three segments of 5 s-long strips. In this way, there were 1,602 5 s segments of I-AVB, 1,411 segments of PAC, and 1,642 segments of PVC. For LBBB and STE recordings, the data were split into a set of 5 s segments up to eight in turn, which was repeated three times with different start points, the first time started from the first data, the 201st data for the second time, and the 401st data for the third time, respectively. Thus, 1,677 segments of LBBB and 1,896 of STE were obtained.

#### Mapping ECG Signals Into Texture Images

**FIGURE 3:**
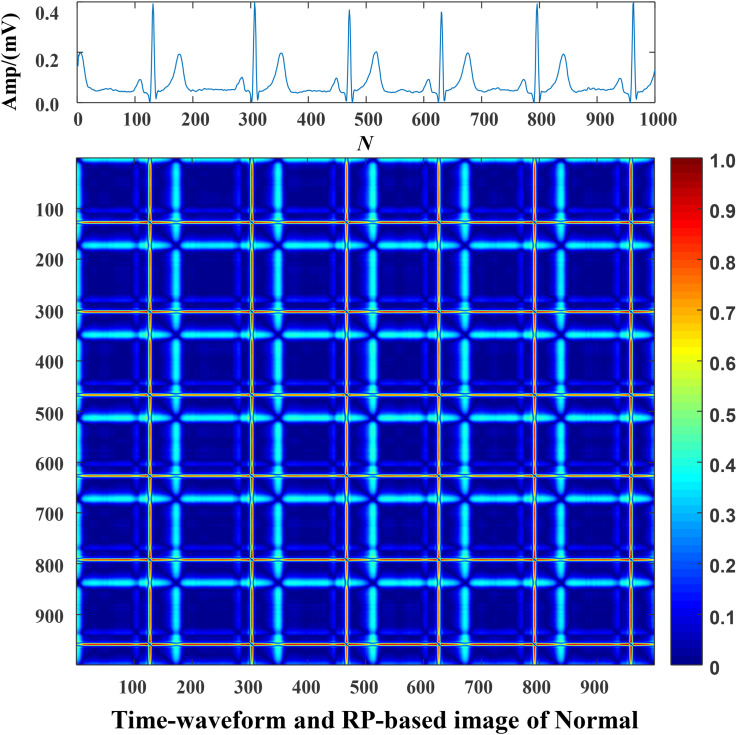
The Normal ECG time waveform and its corresponding RP-based image.

**FIGURE 4:**
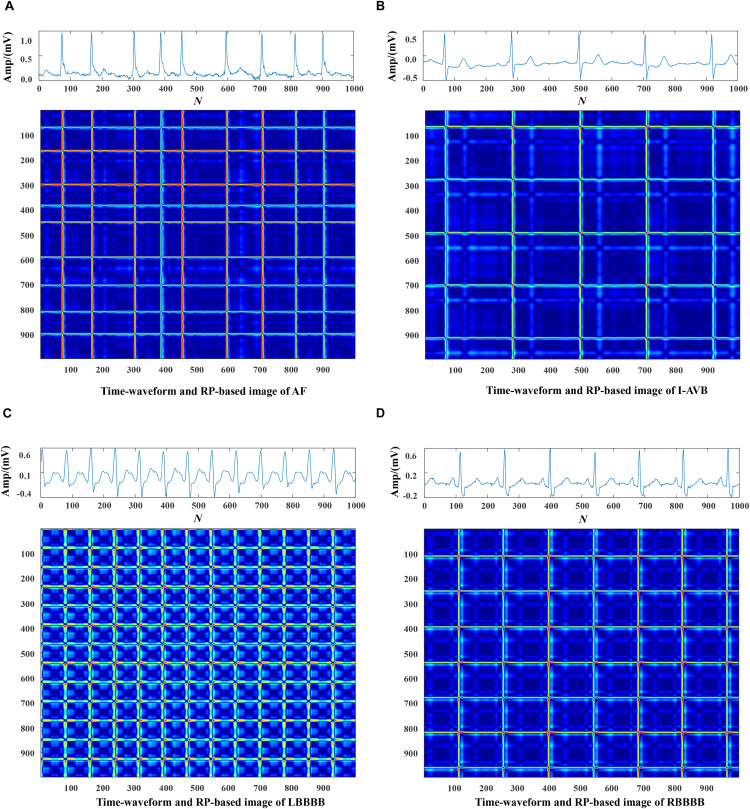
The ECG time waveforms and their corresponding RP-based images of AF, I-AVB, LBBB and RBBB.

**FIGURE 5:**
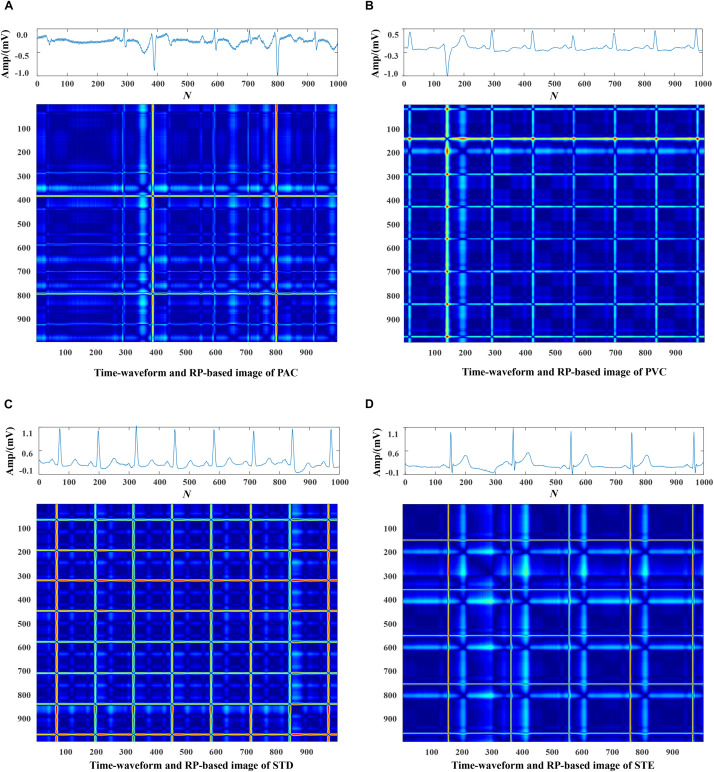
The ECG time waveforms and their corresponding RP-based images of PAC, PVC, STD and STE.

Each data was represented by a set of 5 s ECG strips and further mapped into images through the RP operation. Then, the RP-based images were normalized to the (0–1) range. The CA classification problem was modeled as an image classification task based on RP-based images and CNN. Using a 5 s ECG signal (x) with 1,000 data points, the 2D phase space trajectory is constructed from *x* by the time delay of one point. States in the phase space are shown with bold dots: s_1_ (x_1_, x_2_), s_2_ (x_2_, x_3_), ……, s_999_ (x_999_, x_1000_) (Debayle et al., 2017). The RP R is a 999 × 999 square matrix with *R*_*i,j*_ = dist (s_*i*_, s_*j*_). In Figures 3–5, taking each class signal in the Lead II for instance, the time waveform of ECG recordings with nine types of the classes and corresponding RP-based images were shown. In the RP, different colors can be observed, which are associated with the distance values between plots on the ECG signals. The lowest distance values are coded with a blue color, and the highest distance values are coded with a red color. Moreover, the RP contains textures that are single dots, lines including diagonal, vertical, and horizontal lines, and typology information, including those characterized as homogeneous, periodic, drift, and disrupted. Obviously, there are patterns and information in RP that are not always very easy to see in the time series visually.

### Classification

**FIGURE 6:**
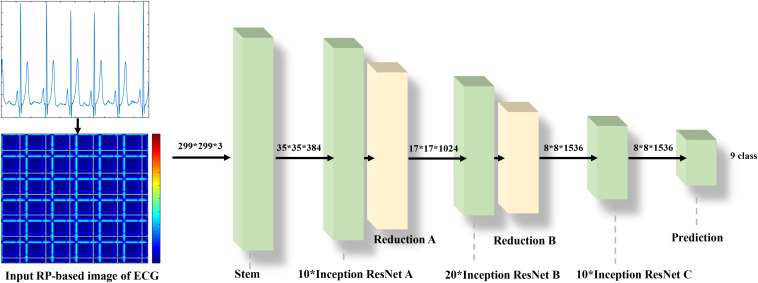
The workflow of the proposed approach for CA classification.

As the flow chart shown in Figure 6, using the RP method, a 2D representation of an ECG time-series signal was obtained and then the 999 × 999 RP-based texture images of 5 s ECG strips were resized to 299 × 299 × 3 and fed into the Inceptive-ResNet-v2 model as input signals. A transfer learning approach was introduced on the generalizability of pre-trained models (Wang et al., 2019). The entire model was trained using Adam optimizer with default parameters, a learning rate of 0.001, and a batch size of 128. Cross entropy loss was calculated for the batched output and corresponding label, and the average gradient was backpropagated to all the weight in the previously mentioned layers. In the process, the training datasets were fed into the network in batches to train models. Then, the model producing the best performance on the validation dataset was selected for further classification on the test dataset. Since the fivefold cross-validation was applied, this process was repeated.

#### Computing Environment

The experiments were performed on Wiener nodes of the University of Queensland computer cluster with 4^∗^ Nvidia Volta V100 SXM2 connected GPU’s per node. Each node contains 5,120 CUDA cores, 640 Tensorflow hardware cores, 32 GB of HBM2 class memory. This model was implemented using the Tensorflow 3.6 and Karas DL framework.

#### Performance of Experiments

To assess whether the proposed method leads to benefits for the CA task, precision, recall, and F1-score were used to evaluate the performance of Inception-ResNet-v2 in typical classification metrics for each class. They were defined as:

(4) $$Precision=\frac{TP}{TP+FP}$$

(5) $$Recall=\frac{TP}{TP+FN}$$

(6) $$F1=\frac{2\left(Precision*Recall\right)}{Precision+Recall}$$

Here, *TP* is the number of data that are correctly classified to a specific class. *FP* is the number of data that are classified to a specific class, but they belonged to other classes. *FN* is the number of data that are misclassified to other classes, but they belonged to a specific class. The average F1-score among classes is computed to evaluate the final performance of the model.

To introduce the RP-based Inception ResNet-v2 method for addressing the CA classification task, this section describes how to find the optimal leads of ECG as the input signal. At first, the full 12-lead RP-based ECG images were fed into the network and obtained an overall average F1-score of 0.7066. In the second step, we did the CA classification based on each single-lead RP-based images as the input signal. The results indicated that lead II was one of the best-performing single leads, followed by the lead V4 and aVR, with the performance ranking first, second, and third in the overall nine types of classification average F1-score of 0.7337, 0.7319, and 0.7313. In the third step, using the above three leads signals, we made different random combinations as the input signals for CA classification. The optimal performance (average F1-score of 0.844) was achieved on the combination of Lead II and lead aVR. These two lead data were divided into several 5 s ECG data segments and then converted into a 2D RP-based image separately. All these images formed into an image dataset as input signals to do the CA classification. To maintain class prevalence between data splits, 20% data of each class were randomly selected as the test set for assessing algorithm performance independently, and 80% data of each type were the training and validation sets. Then, a fivefold stratified split was applied to the training and validation sets. Data four in five were adopted to create a training set, and the other one split as the validation set.

**FIGURE 7:**
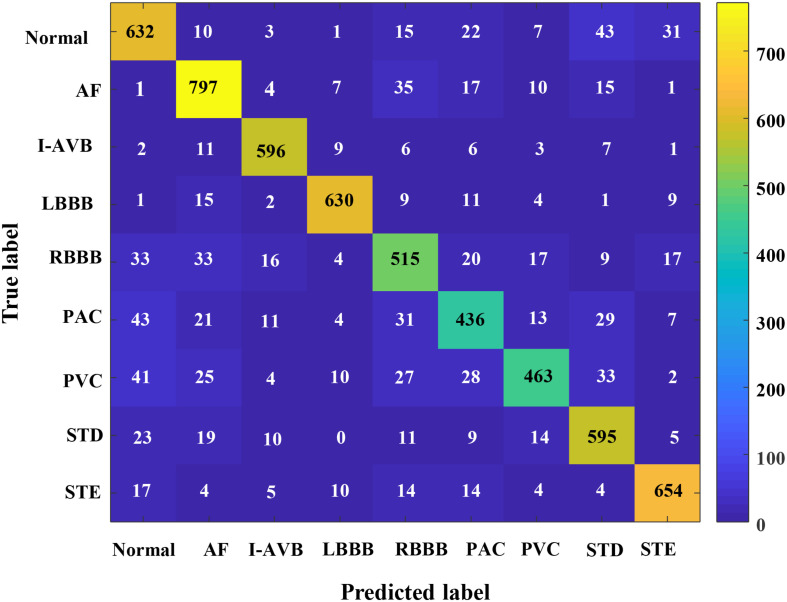
The confusion matrix of the proposed method for CA classification.

In this section, the results concerning the Inception-ResNet-v2 with the RP-based images of Lead II and lead aVR as input data are detailed in Table 2. The proposed method achieved an overall F1-score of 0.844 from the fivefold cross-validation experiments. The average precision is 0.847, and the average recall is 0.847 for the nine classifications of the CA using only two leads of the recordings. Besides, the highest prediction accuracy F1-score in nine classes was obtained at LBBB (0.929) followed by I-AVB (0.923), while prediction for PAC has the lowest F1-score (0.753). In Figure 7, the confusion matrix of the proposed method was drawn. It outlined the data of predictions for each class. There is a relatively small error between Normal rhythm and AF, I-AVB, and LBBB, which implies that the Inception-ResNet-v2 was effective in predicting AF, I-AVB, and LBBB based on the RP texture images, while the method had difficulties in distinguishing PAC rhythms from other rhythms.

**TABLE 2:** Classification performance of Inception-ResNet-v2 based on RP images.

CA Type	Precision	Recall	F1-score
Normal	0.797	0.827	0.812
AF	0.852	0.898	0.875
I-AVB	0.916	0.930	0.923
LBBB	0.933	0.924	0.929
RBBB	0.777	0.776	0.776
PAC	0.774	0.733	0.753
PVC	0.865	0.731	0.793
STD	0.808	0.867	0.837
STE	0.899	0.901	0.900
Avg/total	0.847	0.847	0.844

#### Comparison With Reference Models

To explore the reliability and effectiveness of the proposed method, we implemented seven state-of-the-art models, including Xception (Chollet, 2017), Resnet 50 (He et al., 2016), Resnext (Saining Xie et al., 2017), Densenet (Gao et al., 2017), Inception-ResNet-v1, Inception-v3, and Inception-v4 (Szegedy et al., 2016) as reference. The data augmentation and regularization strategies of training and testing sets are provided separately to ensure that the results of different algorithms are comparable. The same hyperparameters, including learning rate and batch size, were used for the proposed and all reference models. For comparison, the average F1-score of each class was calculated for each architecture. The results of each model can be found in Table 3.

**TABLE 3:** Classification performance of different reference models.

Type		F1-score					
	Xception	Resnet 50	Densenet	Resnext	Inception-ResNet-v1	Inception-v3	Inception-v4	Inception-ResNet-v2
Normal	0.77	0.76	0.75	0.75	0.78	0.72	0.73	0.812
AF	0.85	0.85	0.85	0.88	0.86	0.86	0.85	0.875
I-AVB	0.88	0.88	0.87	0.88	0.89	0.87	0.87	0.923
LBBB	0.89	0.90	0.92	0.91	0.93	0.91	0.91	0.929
RBBB	0.70	0.72	0.70	0.72	0.72	0.71	0.71	0.776
PAC	0.67	0.65	0.64	0.68	0.64	0.60	0.58	0.753
PVC	0.72	0.72	0.71	0.74	0.76	0.68	0.65	0.793
STD	0.79	0.79	0.76	0.79	0.79	0.77	0.75	0.837
STE	0.85	0.83	0.86	0.85	0.90	0.87	0.86	0.900
Avg/total	0.80	0.79	0.79	0.80	0.81	0.78	0.77	0.844

The comparison highlights that for CA based on RP texture images, the Inception-ResNet-v2 achieved an average F1-score of 0.844, which was higher than other classification frameworks. It was shown that the Inception-ResNet-v2 outperformed Xception, Resnet50, Resnext, Inception V3, and Inception V4 in the F1-score of all classes and almost outperformed Densenet in all classes except for one LBBB class where two models performed comparably (F1-score 0.92). Moreover, the Inception-ResNet-v1 achieved an average F1-score of 0.81, with the optimal performance of the prediction on LBBB and STE class. In identifying the LBBB class, almost all the models achieved significantly higher F1-scores compared with other classes. However, all the models had the lowest F1-score in the prediction of the PAC class.

#### Comparison of RP-Based With Other Image-Based Methods for CA Classification

In this work, for CA classification, we also compared the RP method with two other traditional methods that transform the ECG signal to 2D images, including the Wavelet time-frequency images and the time waveform. In this section, the continuous wavelet transform (CWT) method (He et al., 2018) was used to transform the ECG time-domain signals, each of which has a duration of 5 s (1,000 sample points given the sampling rate of 200 Hz), into time-frequency domain signals with six continuous wavelet functions including Complex Gaussian wavelets (cgau8), Complex Morlet wavelet (cmor), Frequency B-Spline wavelets (fbsp), Gaussian wavelets (gaus8), Mexican hat wavelet (mexh), and Morlet wavelet, resulting in the 2D time-frequency representation of the segmented ECG recordings. The proposed Inception-ResNet-v2 was properly trained to process the CA classification. For the time waveform, the segmented 5 s ECG recordings of each class were plotted, and then the waveforms of the time series were used as input sets. These two kinds of 2D images and the RP-based 2D images were fed into the Inception-ResNet-v2 network to process the ECG classification, respectively.

It is observed that the proposed RP method obtained the best average F1-score (0.844) far over the performance of the time waveform (0.70) and that of the Wavelet time-frequency input signals (below 0.70), as shown in Table 4. In identifying the LBBB class, the model achieved the highest F1-scores compared with other classes in the time waveform database and the RP-based images, while the cgau8 and fbsp achieved the highest F1-score of 0.82 and 0.81 at the I-AVB class, respectively. Moreover, in the prediction of the PAC class, these three databases all obtained a poor F1-score. Additionally, the model based on the Wavelet time-frequency images performed poorly than the other two kinds of input signals.

**TABLE 4:** Classification performance of different 2D images-based input data.

Type	F1-score							
	Wavelet time-frequency images						Time waveform	RP-based images
	Cgau8	Cmor	Fbsp	Gaus8	Mexh	Morl		
Normal	0.56	0.56	0.57	0.57	0.61	0.56	0.61	0.812
AF	0.76	0.77	0.75	0.81	0.75	0.77	0.80	0.875
I-AVB	0.82	0.80	0.81	0.77	0.80	0.78	0.81	0.923
LBBB	0.71	0.74	0.71	0.68	0.67	0.72	0.87	0.929
RBBB	0.56	0.53	0.57	0.54	0.54	0.53	0.60	0.776
PAC	0.49	0.39	0.43	0.40	0.43	0.44	0.52	0.753
PVC	0.58	0.56	0.56	0.55	0.56	0.52	0.58	0.793
STD	0.60	0.63	0.62	0.64	0.61	0.61	0.62	0.837
STE	0.53	0.41	0.51	0.44	0.47	0.56	0.81	0.900
Avg/total	0.63	0.61	0.62	0.62	0.62	0.61	0.70	0.844

#### Comparison of the Proposed Method With Other Published Works for CA Classification Based on the 1st China Physiological Signal Challenge 2018 Dataset

In this part, we compared the proposed 2D RP-based method with 1D ECG-based works in literature based on the same public dataset (1st China Physiological Signal Challenge 2018 dataset). Table 5 presents the F1-score on each type of CA and the average F1-score of the nine classes based on the different methods. It contains two parts, including the top three ranks in the challenge and comparison of the proposed method with methods reported. He et al. (2019) ranked first place and achieved an average F1-score for nine classes of 0.799 based on the publicly released dataset. Chen et al. (2020) ranked third place and obtained an F1-score of 0.797. Yao et al. (2019) achieved an average F1-score of 0.812. The results suggested that our proposed RP-based method reached the average nine-class F1-score of 0.844 (excellent performance), which outperformed others. Besides, on the classification of I-AVB, LBBB, STD, and STE, the proposed method achieved a better F1-score than other works. Table 6 presents detailed information, including the input signal, ECG leads, performance, and networks used by the different approaches mentioned above. Table 6 suggested the superiority of the proposed 2D RP approaches: the proposed method introduced the RP-based 2D images as input signals, while others all used the 1D ECG time series; in this study, we used few leads (only two of the 12-lead original ECG data); the proposed method achieved the highest average F1-score of 0.844, although with more trainable parameters than other 1D models.

**TABLE 5:** The performance of the published 1D ECG-based works and the proposed method.

Rank	Team	Normal	AF	I-AVB	LBBB	RBBB	PAC	PVC	STD	STE	Avg/total
**The hidden test set of the 1st China Physiological Signal Challenge 2018 F1-score**											
1	He et al.	0.748	0.920	0.882	0.889	0.883	0.787	0.851	0.780	0.780	0.836
2	Cai et al.	0.765	0.927	0.887	0.886	0.880	0.812	0.800	0.784	0.753	0.833
3	Chen et al.	0.752	0.930	0.871	0.915	0.839	0.832	0.833	0.800	0.667	0.823
**The publicly released dataset of the1st China Physiological Signal Challenge 2018 F1-score**											
	Yao et al.	0.789	0.920	0.850	0.872	0.933	0.736	0.861	0.789	0.556	0.812
	He et al.	0.755	0.846	0.870	0.869	0.780	0.751	0.829	0.790	0.704	0.799
	Chen et al.	0.795	0.897	0.865	0.821	0.911	0.734	0.852	0.788	0.509	0.797
	RP-based	0.812	0.875	0.923	0.929	0.776	0.753	0.793	0.837	0.900	0.844

**TABLE 6:** Comparison of the published 1D ECG-based works with the proposed method.

Team	Input signal	ECG leads	Network	Parameters	Avg/total F1-score
Yao et al.	1D ECG	12 leads	ResNet+BiLSTM-GMP	4,984,640	0.812
He et al.	1D ECG	12 leads	ATI-CNN	No report	0.799
Chen et al.	1D ECG	12 leads	CNN+BRNN+Attention	28,035	0.797
RP-based	2D RP images	2 leads (lead II and aVR)	Inception-resnet-v2	46,964,673	0.844

#### Testing the Generalization Ability of the Proposed Method Using Some Other Databases

In this section, three datasets of the PhysioNet/Computing in Cardiology Challenge 2020 (Perez Alday et al., 2021) were adopted to evaluate the generalization of the proposed method, as listed in Table 7.

**TABLE 7:** Data profile for the CPSC, PTB_XL, and Georgia ECG dataset.

Database	Sample frequency	Mean duration	Number of subjects					
			Normal	AF	I-AVB	LBBB	RBBB	PAC
CPSC	500 Hz	16.2 s	918	1,221	722	236	1,857	616
PTB_XL	500 Hz	10.0 s	18,092	1,514	797	536	0	398
Georgia	500 Hz	10.0 s	1,752	570	769	231	542	639

CPSC (Liu et al., 2018). The data source is the public training dataset from the China Physiological Signal Challenge (De Chazal et al., 2004).

PTB_XL (Wagner et al., 2020). The source is the Physikalisch Technische Bundesanstalt (PTB), Brunswick, Germany, a large, publicly available ECG dataset.

Georgia. Georgia is a 12-lead ECG Challenge Database (Emory University, Atlanta, GA, United States) representing a large population from the Southeastern United States.

The sampling rate of the signal is 500 Hz. In this experiment, the PTB_XL includes five classes (Normal, AF, I-AVB, LBBB, and PAC), and the CPSC and Georgia contain six types (Normal, AF, I-AVB, LBBB, RBBB, and PAC). The lead II and lead aVR of each ECG data in these datasets were used as the input signal for CA classification.

Each signal was resampled at 200 Hz and then segmented into two 5 s long patches and mapped into RP-based 2D images with a normalized range (0–1). These 2D images were input signals of the network for classification. The results suggested that the proposed method achieved an average F1-score of 0.8521 on CPSC, 0.8529 on Georgia, and 0.8862 on PTB_XL in Table 8. Moreover, the performance of the proposed method on the PTB_XL is the best, and the high prediction F1-scores were obtained at Normal (0.9417) and LBBB (0.9246); in contrast, prediction for PAC has the lowest *F*-score (0.7832). For Georgia, the proposed method can effectively classify the AF, I-AVB, LBBB, and PAC. These results highlighted that the proposed method had excellent CA classification performance and generalization ability.

**TABLE 8:** Classification performance of different ECG datasets.

Database	Avg/total F1-score	Classification of subjects F1-score					
		Normal	AF	I-AVB	LBBB	RBBB	PAC
CPSC	0.8521	0.7905	0.9269	0.8921	0.8825	0.8942	0.7266
PTB_XL	0.8862	0.9417	0.9167	0.8644	0.9246	0	0.7837
Georgia	0.8529	0.9237	0.8197	0.8706	0.8767	0.8629	0.7639

### Discussion

This work intends to study whether the RP method is appropriate for the DL-based CA classification. To represent features that are not easy to be observed in the time domain, we transformed the ECG signals into 2D RP-texture images for the CNN-based CA classification. In the experiments, the results showed that different CNN models effectively learned the information based on the RP input images in the training process. Moreover, the Inception-ResNet-v2 network achieved the optimal performance with an average F1-score of 0.844, followed by the Inception-ResNet-v1 network of 0.81. It is explained that the Inception-ResNet module contains multiple filters of various sizes, capturing the RP image spatial features in different scales. Besides, larger filters may be more effective due to the increased perceptive field being able to more effectively account for the variations of the signal over time.

To analyze whether the RP-based method performs better in the CA classification than other methods, we compared the time-waveform and Wavelet time-frequency images with RP-based images as input signals, respectively. The results showed that the RP-based model achieved an optimal average F1-score of 0.844, which is better than the time-waveform and Wavelet time-frequency images. Moreover, we compared the 2D RP-based method with the published 1D ECG-based works based on the same publicly dataset (the 1st China Physiological Signal Challenge 2018 dataset). The results indicated that the proposed 2D RP-based approach outperforms 1D signal-based models in the CA classification even with only two of 12 leads. The RP method could visualize certain aspects of the 2D phase space trajectory, extracting inherent texture features between different points of an ECG recording (Ouyang et al., 2008, 2014). It highlights that the RP-based method has a high potential to improve the CA classification accuracy with the CNN framework.

Three ECG datasets of the PhysioNet/Computing in Cardiology Challenge 2020 were adopted to study the generalization ability of the proposed method. The proposed method achieved an average F1-score of 0.8529 on Georgia, 0.8521 on CPSC, and 0.8862 on PTB_XL. The results showed that the 2D RP-based CA classification method has excellent generalization ability.

The other contribution of our work is to find the optimal ECG leads of the nine types of CA classification. Excellent classification results were obtained with lead II and lead aVR. We found that the network could successfully process the CA classification without access to the full 12 leads data. It is known that, among the 12 leads, lead II offers physician-favored signal, and lead aVR may reflect atrial and ventricular information from the right upper side of the heart (Gorgels et al., 2001). Chen et al. (2020) reported that aVR was one of the best-performance single leads in the classical CA classification experiments. In our study, it is also confirmed that the RPs of these two-lead signals are useful for the machine-learning-based CA classification. Besides, RP-based lead II and lead aVR ECG recordings performed differently in various classes. As shown in Table 2, the best prediction is for the LBBB (F1-score 0.929), followed by I-AVB (F1-score 0.923), while the prediction of the PAC was relatively poor. This may be due to the fact that the PAC occurs when a focus in the atrium (not the sinoatrial node) generates an action potential before the next scheduled SA node action potential, which is complex and less common. This makes it challenging to differentiate them from PVC, normal, and other arrhythmias (Surawicz et al., 2009). A similar finding has also been reported in early works (Yao et al., 2019; Chen et al., 2020).

This work studied the classification of CA based on the 1st China Physiological Signal Challenge 2018 ECG dataset. Further studies will be required to investigate those involving technical problems such as data imbalance, and the RP method will be refined to improve the prediction of PAC.

## Conclusion

In this paper, we proposed a DL-based method for automatic CA classification. In this method, the RP-based 2D texture images are processed as input data, which contain rich features unobservable from the standard time-domain and time-frequency domain. Based on RP-texture images, the Inception-ResNet-v2 network was used to predict and classify various CAs. In our study, the proposed method offers excellent performance with only two-lead ECG data without accessing the full 12-lead ECG recordings. It implies that this RP-based CA classification method may have the potential to be used as a diagnostic tool in conditions/places where access to a 12-lead ECG is difficult.

## Data Availability Statement

The publicly released dataset of the First China Physiological Signal Challenge 2018 is available at http://2018.icbeb.org/Challenge.html.

## Author Contributions

FL conceived the study. HZ performed the design and implementation of the work. CL helped with the experiment design. ZZ helped with the implementation of the recurrent plot. YX and RD contributed to the discussion of the ECG. XL and YH helped the algorithm design of Resnet 50. HZ wrote the manuscript. FL, CL, and LX helped to review and improve the manuscript. All authors read and approved the final manuscript.

## Conflict of Interest

The authors declare that the research was conducted in the absence of any commercial or financial relationships that could be construed as a potential conflict of interest.
